# Expression of Concern: The effect of dietary lipid manipulation on murine splenic lymphocytes apoptosis and heat shock protein over expression

**DOI:** 10.1093/femspd/ftaf008

**Published:** 2025-08-29

This is an expression of concern regarding: C. Romano Carratelli, I. Nuzzo, T. Vitiello, E. Galdiero, F. Galdiero, The effect of dietary lipid manipulation on murine splenic lymphocytes apoptosis and heat shock protein over expression, *FEMS Immunology & Medical Microbiology*, Volume 24, Issue 1, May 1999, Pages 19–25, https://doi.org/10.1111/j.1574-695X.1999.tb01260.x

Concerns regarding similarities within Figure 1 were raised on Pub Peer [https://pubpeer.com/publications/BC31F6879C23E32133B403250BF675] and subsequently were flagged to the Editor-in-Chief in 2025.

The journal attempted to contact the corresponding author but has not received a response and is investigating our concerns about Figure 1 in line with COPE guidance. The journal is publishing this Expression of Concern to notify readers of the concerns whilst the outcome of the investigation is pending and advises readers to examine the details of this study with particular care.

